# Instructive percutaneous coronary intervention to avoid the risk of side branch occlusion at a lesion with a lotus root appearance: a case report

**DOI:** 10.1186/s12872-016-0335-y

**Published:** 2016-08-02

**Authors:** Tetsuya Nomura, Taku Kato, Hiroshi Kubota, Daisuke Miyawaki, Ryota Urata, Takeshi Sugimoto, Yusuke Higuchi, Natsuya Keira, Tetsuya Tatsumi

**Affiliations:** Department of Cardiovascular Medicine, Nantan General Hospital, 25, Yagi-Ueno, Yagi-cho, Nantan City, Japan

**Keywords:** Case report, Lotus root appearance, Optical coherence tomography, Dual lumen microcatheter, Bifurcation, Side branch

## Abstract

**Background:**

A lotus root appearance is a rare entity, and there is little opportunity to perform coronary intervention for this kind of lesion. Because of its peculiar anatomical characteristics, one of the problems regarding percutaneous coronary intervention (PCI) for these lesions is related to the involvement of branch vessels.

**Case presentation:**

We encountered a case of PCI for a stenotic lesion with a lotus root appearance in the mid-portion of the right coronary artery (RCA). To avoid the risk of right ventricular (RV) branch occlusion due to stent deployment in the main RCA, we re-crossed the third guidewire into the main RCA via the nearest point to the RV branch ostium through the communicating vascular lumen. Thereafter, we deployed a drug-eluting stent in the main RCA crossing over the RV branch, and the ostium of the RV branch remained intact, as we expected.

**Conclusions:**

This case is the first report in the world describing the details of how to maintain the patency of the side branch bifurcating from a lesion with a lotus root appearance under optical coherence tomography guidance.

## Background

A lotus root appearance is a rare entity that can be detected by intravenous ultrasound sonography (IVUS) or optical coherence tomography (OCT) during the daily practice of coronary catheterization. Lesions with a lotus root appearance are characterized by multiple vascular channels separated by wall partitions, communicating with each other and converging into a single lumen at proximal and distal sites. The detailed origin of the lotus root appearance is still unknown. Although there is little opportunity to perform coronary intervention for this kind of lesion, we always have to be aware of how to maintain the patency of a side branch (SB) bifurcating from a lesion with a lotus root appearance.

## Case presentation

**Fig. 1:**
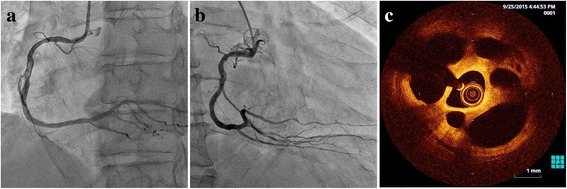
**a** A control image demonstrates a moderate stenotic lesion with some linear opacity in the mid-portion of the RCA. **b** The RV branch bifurcates from the stenotic lesion. **c** A typical image of a lotus root appearance is observed for the full length of the stenotic lesion

A 70-year-old male was admitted to our hospital complaining of exertional chest pain on effort. His coronary risk factor was only hypertension. A twelve-lead electrocardiogram at rest demonstrated a normal sinus rhythm and no significant ST-segment change. Ultrasound echocardiography showed slight motion abnormality in the inferior wall. Coronary computed tomography angiography revealed an image which resembled coronary dissection in the mid-portion of the right coronary artery (RCA). Therefore, we performed cardiac catheterization with a left trans-radial approach, and a moderate stenotic lesion with some linear opacity was observed there (Fig. 1a). Also, the right ventricular (RV) branch bifurcated from the stenotic lesion (Fig. 1a,b). Considering his clinical background and the findings of the examinations, we decided to perform percutaneous coronary intervention (PCI) for this lesion in the mid RCA.

**Fig. 2:**
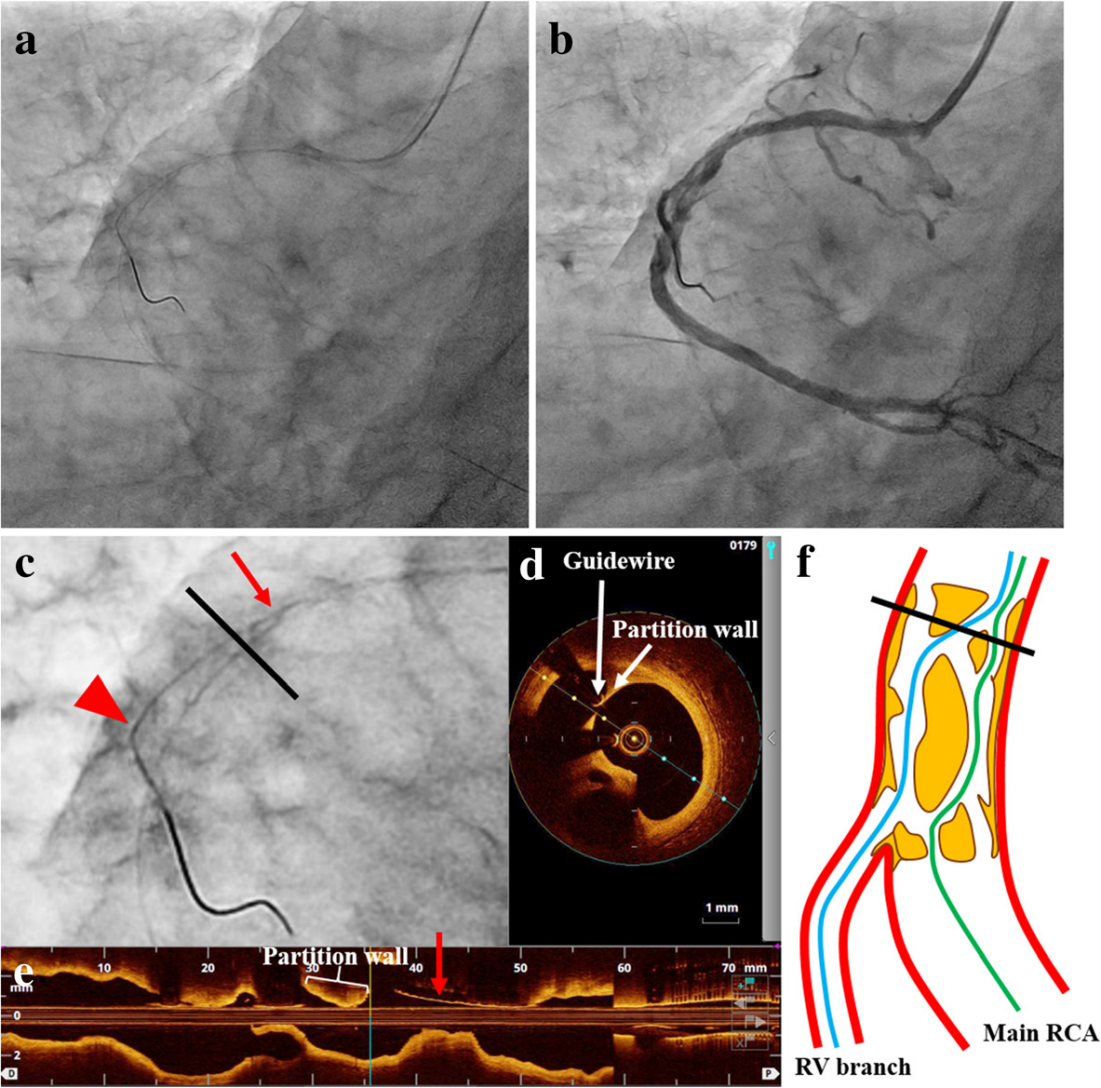
**a**, **b** Two guidewires initially pass through both the main RCA and RV branch. **c** The magnified image around the lesion shows the difference between the positions of the two guidewires in the RCA. The *red arrow* indicates the point where the guidewire branches off toward the RV branch. The *red arrowhead* indicates the true bifurcation point. **d**, **e** The OCT findings clearly show that the guidewire branches off toward the RV branch from a point more proximal than the true bifurcation point. **d** is a cross-sectional image at the distance indicated with the *black bar* in **c**, **f**. **f** The outlook of the lesion with the lotus root appearance after initial guidewire passage. *Blue* and *green lines* indicate the guidewires passing into the RV branch and main RCA, respectively

**Fig. 3:**
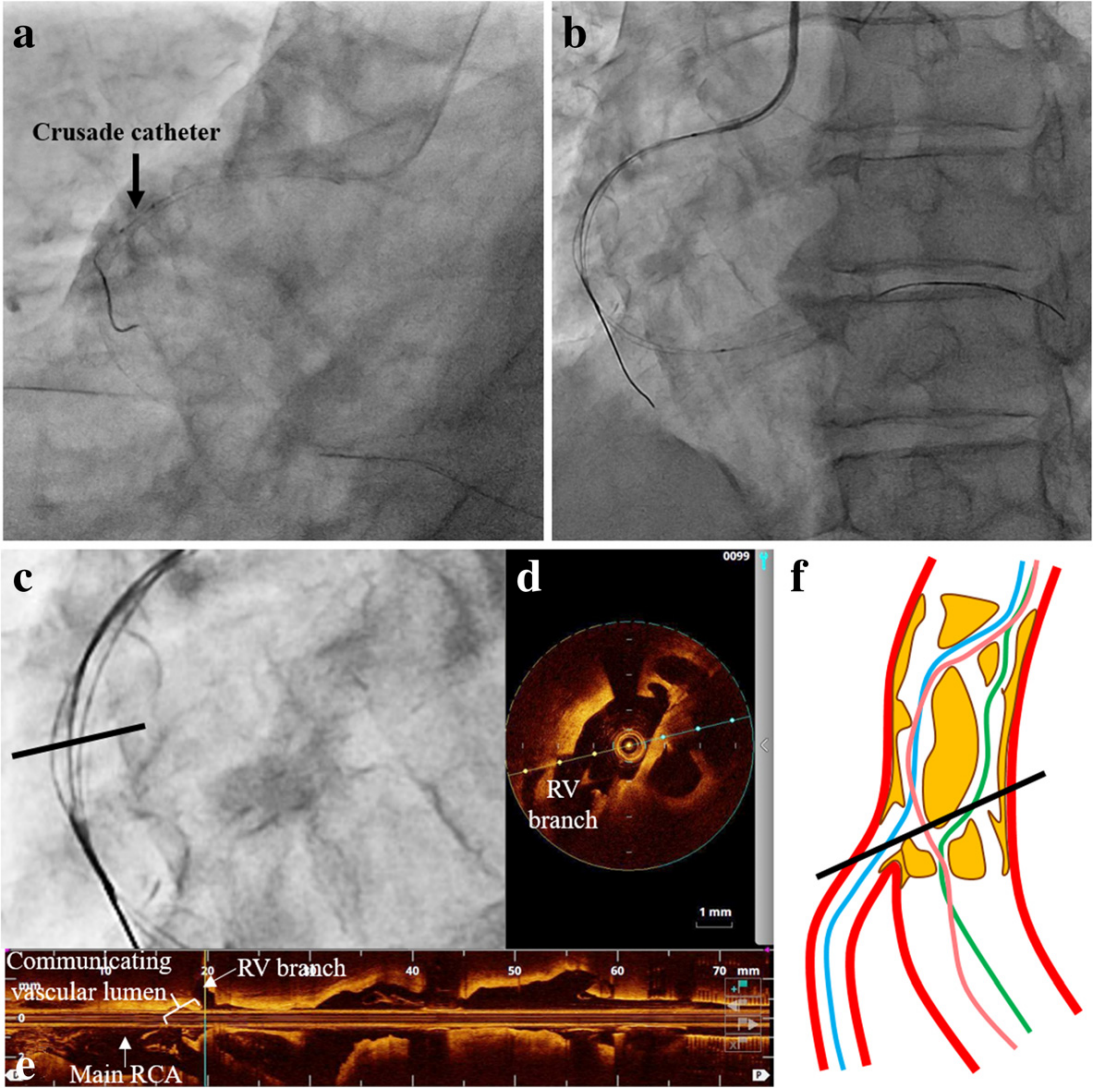
**a**, **b** An attempt is made to pass the Fielder FC guidewire toward the main RCA via the true bifurcation point using the Crusade catheter. **c** The magnified image around the re-crossing point of the third guidewire shows the three different courses of the guidewire-passing routes. **d**, **e** The OCT findings clearly show the third guidewire crossing from the nearest point of the RV branch ostium to the distal RCA through the communicating vascular lumen. **d** is a cross sectional image at the distance indicated with the *black bar* in **c**, **f**. **f** The outlook of the lesion with the lotus root appearance after the third guidewire re-crossing. *Blue* and *green lines* indicate the initial guidewires passing into the RV branch and main RCA, respectively. The pink line indicates the third guidewire

A 6Fr Sherpa NX ACTIVE JR4 guiding catheter (Medtronic Inc., MN, USA) was engaged in the RCA ostium, and a Sion blue guidewire (Asahi Intecc Co., Ltd., Aichi, Japan) was advanced along the side of the greater curvature of the RCA toward the distal RV branch. Then, we tried to introduce the Sion guidewire (Asahi Intecc) along the side of the lesser curvature of the RCA toward the distal RCA (Fig. 2a-c). We performed OCT scanning with a Dragonfly imaging catheter (St. Jude Medical, Inc., MN, USA) around the lesion, and typical pictures of a lotus root appearance were observed along the full length of the stenotic lesion (Fig. 1c). The OCT findings also clearly showed that the Sion blue guidewire branched off toward the RV branch from a point (Fig. 2c,e*Red arrows*) more proximal than the true bifurcation point (Fig. 2c, *Arrowheads*) and some partition walls existed between the two guidewires (Fig. 2d,e). Therefore, we tried to re-cross the new third guidewire into the main RCA before deploying the stent. For that purpose, we used the Crusade catheter (KANEKA Corp., Osaka, Japan), a dual lumen microcatheter, mounted on the Sion blue guidewire in the RV branch, and re-crossed the third guidewire Fielder FC guidewire (Asahi Intecc) through the over-the-wire lumen of the Crusade catheter toward the main RCA via the nearer point to the RV branch ostium as close as possible (Fig. 3a). After re-crossing the third guidewire to the distal RCA, we checked the OCT mounted on this third guidewire (Fig. 3b-e). It was verified that the third guidewire crossed from the nearest point of the RV branch ostium to the distal RCA through the communicating vascular lumen (Fig. 3d-f).

**Fig. 4:**
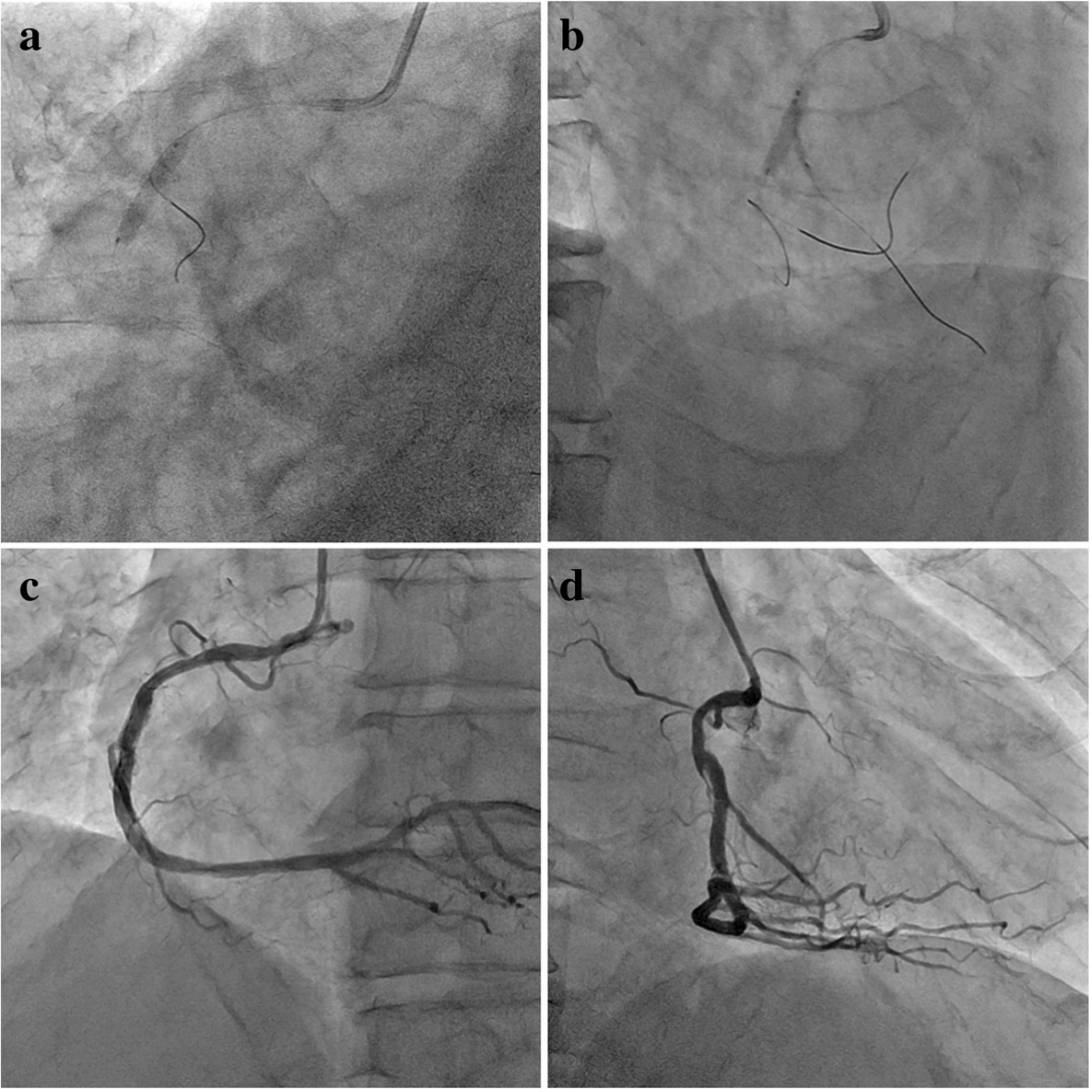
**a** A Resolute Integrity drug-eluting stent is deployed in the main RCA crossing over the RV branch. **b** Kissing balloon inflation using two semi-compliant balloons is performed. **c**, **d** The final angiogram demonstrates favorable dilation of the lesion in the mid RCA with good patency of the RV branch

We inflated the scoring balloon NSE ALPHA (GOODMAN Co., Ltd., Aichi, Japan) with a 2.5-mm diameter and then deployed a Resolute Integrity drug-eluting stent (Medtronic) of 3.5/22 mm in the main RCA crossing over the RV branch (Fig. 4a). After re-wiring with the Sion guidewire through the stent strut to the RV branch with the Crusade catheter, we completed the procedure with kissing balloon inflation using a TREK balloon catheter (Abbott Laboratories, IL, USA) with a 3.5-mm diameter in the main RCA and Kamui balloon catheter (Asahi Intecc) with a 2.0-mm diameter in the RV branch (Fig. 4b). We inflated both balloon catheters to a pressure of 10 atmospheres. Finally, we performed proximal optimization therapy using a Hiryu balloon catheter (Terumo Corp., Tokyo, Japan) with a 4.0-mm diameter at the proximal lesion in the stent. The final angiogram demonstrated favorable dilation of the lesion in the mid RCA with good patency of the RV branch (Fig. 4c,d).

His chest symptom fully resolved after this intervention and no adverse events have been observed since then. We performed follow-up coronary angiography (CAG) at 8-month after PCI. Favorable blood flow was observed in both the main RCA and the RV branch, and OCT findings showed better strut apposition and coverage with neointimal formation.

## Discussion

We encountered a patient who required coronary intervention for the mid-portion of the RCA with a lotus root appearance detected during a survey with OCT. A lotus root appearance was firstly reported as a lesion composed of multiple channels observed by IVUS in a young patient who had a history of suspected Kawasaki disease in 2002 [1]. Although the detailed origin of the lotus root appearance is still unknown, “arteries within the artery” was first described as spontaneous recanalization after coronary thrombotic events in a patient with Kawasaki disease [2], and it is believed to be identical to a lotus root appearance.

A lesion with a lotus root appearance is characterized by multiple vascular channels separated by wall partitions, communicating with each other and converging into a single lumen at proximal and distal sites. As the OCT has become widely used, more and more cases with this kind of lesion have been reported not only in coronary arteries [3, 4] but also in carotid arteries [5]. However, not so many cases have received PCI for this kind of lesion. Several cases verified the validity of performing PCI for these lesions based on a significant decrease of the fractional flow reserve value [6, 7]. Functionally significant ischemic findings are often demonstrated at a lesion with a lotus root appearance, whereas the stenosis rate at that lesion angiographically shows a moderate degree. The reason for the “visual -functional mismatch” is suggested from the OCT images in which the dead end of the majority of multiple intraluminal channels except a skinny channel can cause markedly limited coronary flow.

It is considered that one of the problems regarding PCI for these lesions is related to the involvement of the branch vessels. A previous case report described a strategy to prevent occlusion of the SB which bifurcated from a lesion with a lotus root appearance [8]. In this case report, the operator confirmed the route through which the guidewire passed from the main vessel (MV) to the SB using IVUS. However, the authors did not describe the details about the methods to cross the guidewire into the SB through the optimal branch ostium at the lesion with the lotus root appearance. Similarly in our case, we were concerned about the risk of SB occlusion after stenting in the MV. To avoid this problem, we considered it important to deposit the guidewire using the optimal route. From the findings of OCT, the point at that the vascular lumen most distally branching from the MV was thought to be the true bifurcation point. The Sion blue guidewire branched off toward the RV branch from a point more proximal than the true bifurcation point, and some partition walls existed between the two guidewires (Fig. 2f). Therefore, to avoid SB occlusion due to deploying a stent in the MV, we had to re-cross the guidewire using the optimal route in the main RCA via the nearest point to the RV branch ostium.

**Fig. 5:**
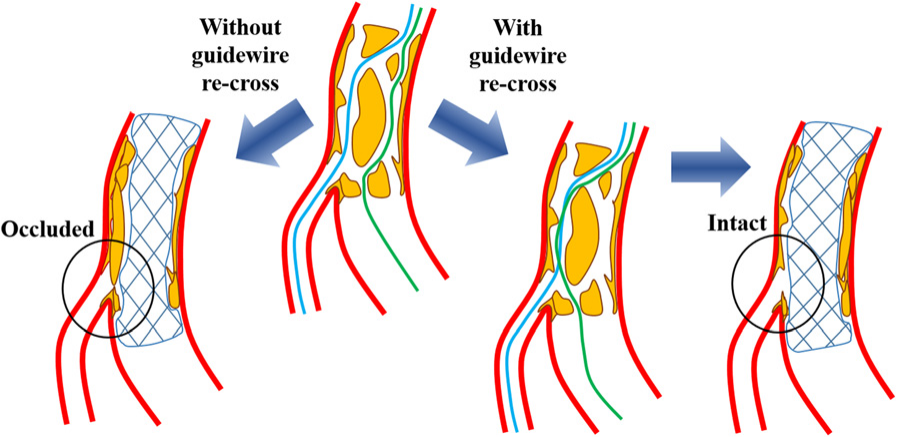
The schema demonstrates the outlook after the deployment of a stent at the lesion with the lotus root appearance with or without guidewire re-cross. Refer to the text for details

Regarding the guidewire re-cross, there were two choices which direction we should take either from the main RCA to the RV branch or its opposite direction. Because the vascular lumens are intricately connected with each other around the bifurcation point in the main RCA and the lumen size at just distal site of the bifurcation in the RV branch was smaller than that in the main RCA, we thought it was more difficult to re-cross a guidewire from the main RCA to the RV branch. Therefore, we adopted a strategy to manipulate the third guidewire with the Crusade dual lumen microcatheter mounted on the guidewire passing into the RV branch (Fig. 3f). If we deploy a stent in the main RCA without re-crossing the guidewire, the RV branch may be occluded with a high probability due to compression of the partition walls toward the ostium of the RV branch. On the other hand, in the case of third guidewire re-crossing from the side of the RV branch to the MV, the partition walls are compressed to the opposite side of the vascular wall from the RV branch ostium by deploying a stent in the MV (Fig. 5). Actually the ostium of the RV branch was intact after deploying a stent, as we expected.

We performed all PCI procedures with the guidance of OCT. The high resolution of OCT remarkably helped us to clearly detect the guidewire position in the lesion with a lotus root appearance. Moreover, OCT is also very useful for characterization of stent healing and vascular responses in the chronic phase as well as accurate procedures in PCI. As shown in recent systemic review, we confirmed better strut apposition and coverage with neointimal formation after zotarolimus-eluting stent implantation in the follow-up CAG [9].

In this case, we took advantage of the Crusade dual lumen microcatheter when introducing the third guidewire along the optimal route. The dual lumen microcatheter allows the operator to deliver the second guidewire through the over-the-wire lumen into the same vascular lumen where the first guidewire exists. Because the vascular lumens are intricately connected with each other around the lesion with a lotus root appearance, we think the dual lumen microcatheter is optimal for this kind of lesion. The dual lumen microcatheter is a very useful device for multipurpose use in practical PCI. It can help us to perform complex PCI procedures more safely, speedily, and steadily.

## Conclusions

A lotus root appearance is a rare entity detected by IVUS or OCT during the daily practice of coronary catheterization. Although there is little opportunity to perform coronary intervention for a lesion with a lotus root appearance, we always have to pay attention to how to maintain the patency of the SB bifurcating from a lesion with a lotus root appearance.

## Abbreviations

CAG, coronary angiography; IVUS, intravenous ultrasound sonography; MV, main vessel; OCT, optical coherence tomography; PCI, percutaneous coronary intervention; RCA, right coronary artery; RV, right ventricular; SB, side branch
